# The Clinicopathologic and Prognostic Significance of Gross Classification on Solitary Hepatocellular Carcinoma After Hepatectomy

**DOI:** 10.1097/MD.0000000000001331

**Published:** 2015-08-14

**Authors:** Jian He, Jiong Shi, Xu Fu, Liang Mao, Tie Zhou, Yudong Qiu, Bin Zhu

**Affiliations:** From the Department of Radiology, Nanjing Drum Tower Hospital, The Affiliated Hospital of Nanjing University Medical School, Jiangsu Province, China (JH, BZ); Department of Pathology, Nanjing Drum Tower Hospital, The Affiliated Hospital of Nanjing University Medical School, Jiangsu Province, China (JS); and Department of Hepatopancreatobiliary Surgery, Nanjing Drum Tower Hospital, The Affiliated Hospital of Nanjing University Medical School, Jiangsu Province, China (XF, LM, TZ, YQ).

## Abstract

The prevalence of hepatitis B virus (HBV) infection is extremely high in China. We aimed to investigate the clinicopathologic and prognostic significance of gross classification on solitary hepatocellular carcinoma (HCC) after hepatectomy.

A total of 144 patients with solitary HCC who underwent hepatectomy were identified retrospectively. Based on the gross appearance, the tumors were divided into single nodular (SN), single nodular with extranodular growth (SNEG), confluent multinodular (CMN), and infiltrative types. Clinicopathologic variables and survival information were compared among patients with those 4 types.

The 144 tumors composed of 25 SN, 34 SNEG, 33 CMN, and 52 infiltrative types. The serum alpha-fetoprotein (AFP) level and HBV infection rate of infiltrative type were significantly higher than other 3 types. The disease-free and overall survival times of infiltrative type were significantly shorter than other 3 types. Univariate and multivariate analysis showed that gross classification, microvascular invasion, and T stage were independent risk factors.

In Chinese patients with solitary HCC, the infiltrative type accounted for a much higher proportion compared with other regions of the world. Infiltrative HCC had higher serum AFP level, HBV infection, and microvascular invasion rates with poorer prognosis compared with other 3 types.

## INTRODUCTION

The gross classification of hepatocellular carcinoma (HCC) was first proposed by Eggel in 1901.^1^ Kanai et al proposed a new gross classification in 1987,^2^ which was accepted by the Liver Cancer Study Group of Japan and is now widely used.^3^ Previous studies have confirmed the prognostic significance of gross classification of HCC in both hepatectomy and living donor liver transplantation (LDLT).^4–8^

It was reported that not only the incidence of HCC, but the gross morphologic features of the tumor varied from one area of the world to another.^9^ Although over half of the new cases of HCC worldwide occur in China,^10^ little has been reported on the gross classification features of HCC in Chinese patients. A HCC with a wholly unclear or ill-defined boundary and infiltrative growth pattern was named infiltrative type, which was seldom referred in previous studies due to its low incidence and resection rate. Since prevalence of chronic hepatitis B virus (HBV) infection is much higher in China and the link between HBV infection and this infiltrating form of HCC is very close,^11^ we hypothesized that infiltrative type would occupy a higher proportion in Chinese patients of HCC.

It was reported that solitary HCC was the most common type, followed by multinodular, diffuse, and massive types.^4,5^ With the progress in imaging techniques, early detection rate of HCC has been improved. And the advances in surgical technique and therapeutic modalities provide the patients with more treatment options. Detailed typing of HCC may optimize the therapeutic effect. So, the purpose of this study was to investigate the gross classification features of solitary HCC in China, and to investigate its clinicopathologic and prognostic significance after hepatectomy.

## METHODS

The procedures followed were in accordance with the ethical standards of the responsible institutional committee on human experimentation and with the Helsinki Declaration of 1975, as revised in 1983. Informed consent from the patient was waived due to its retrospective nature.

### Study Population

From January 2008 to December 2013, 280 consecutive patients with HCCs underwent initial and curative hepatic resections in our institution. Neither macroscopic tumor thrombus nor distant metastasis could be detected pre- and intraoperatively. No preoperative therapy such as transcatheter arterial chemoembolization (TACE), focal ablation, and molecular targeted therapy was performed in those patients.

Inclusion criteria were solitary histologically confirmed HCC, which could be classified according to the gross specimen photographs. A total of 144 cases met the criteria and served as the subjects of this investigation. The other 136 patients were excluded for the following reasons: multifocal lesions (107 cases), loss of gross specimen photographs (24 cases), complete necrosis and inflammatory tissues on pathological findings (5 cases).

### Gross Morphological Types of HCC

**FIGURE 1:**
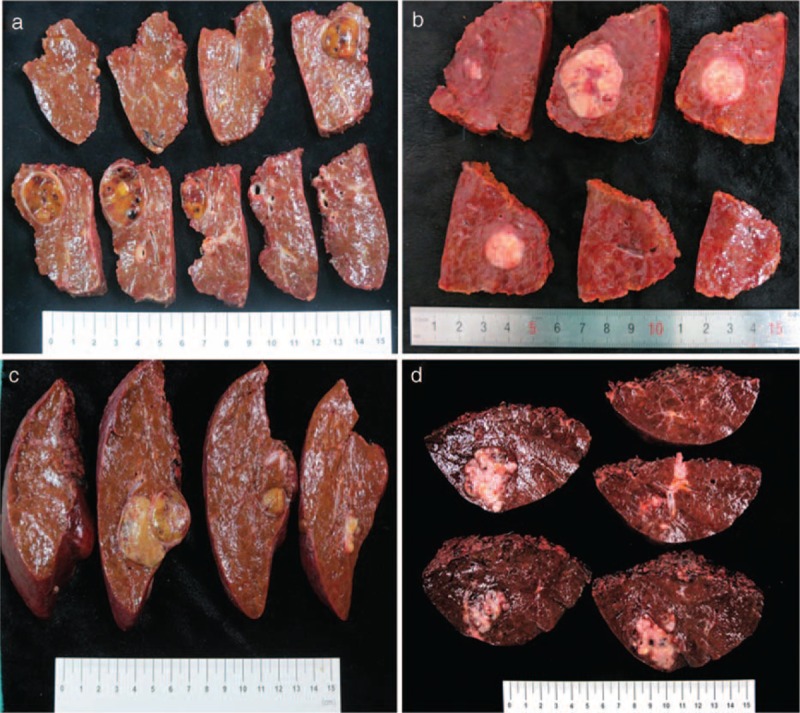
Photographs of hepatocellular carcinoma with different morphological appearance. (A) Type 1, an oval nodule with clear boundary as well as an obvious fibrous capsule; (B) type 2, a roughly oval nodule with a local protrusion at the bottom (arrow); (C) type 3, a lobulated lesion with 2 nodules merging together, each nodule with a clear margin; (D) type 4, a rather irregular lesion with multiple invasion to liver parenchyma.

From all cases, color photographs of the gross surgical specimen were available illustrating the cut surfaces of all slices 10 mm in thickness. Three reviewers (1 pathologist, 1 radiologist, and 1 surgeon) who were blind to the clinical course of the patients read the photographs independently and discussed to achieve consensus if their classifications were inconsistent. HCC lesions were divided into 4 types based on their gross morphological appearance according to the following criteria: type 1, single nodular (SN) type had a round or oval shape with a clear boundary, with or without fibrous pseudocapsule; type 2, single nodular type with extranodular growth (SNEG) roughly resembled type 1 but exhibited local extranodular growth to varying degrees; type 3, confluent multinodular (CMN) type was comprised of a cluster of small and confluent nodules, each nodule with a clear margin or capsule, and the tumor was lobulated as a whole; type 4, infiltrative type had a rather irregular shape and unclear border. Typical examples of the 4 types of HCC were shown in Figure 1.

### Clinicopathologic Review

Demographic information and preoperative laboratory examinations were retrospectively reviewed. Age, gender, serum hepatitis B surface antigen (HBsAg), serum hepatitis C virus antibody (HCV-Ab), the highest preoperative serum alpha-fetoprotein (AFP) values, white blood cell count, platelet count, serum alanine aminotransferase (ALT), aspartate aminotransferase (AST), serum total bilirubin (TB), serum albumin, prothrombin time international normalized ratio (PT-INR), 15-minute retention rate for indocyanine green (ICG R15), and model for end-stage liver disease (MELD) score of each patient were recorded.

All the surgeries were planned and performed by the same group. An anatomic or nonanatomic resection was adopted according to the location, extent of the lesion, and hepatic functional preservation. Anatomic resection included segmentectomy, sectoriectomy, and hemihepatectomy or more, based on Couinaud classification. Nonanatomic resection consisted of limited resection and enucleation.

Microscopic features of each lesion were retrospectively reviewed by 2 pathologists. The histologic grade of tumor differentiation was assigned according to Edmondson–Steiner grading system.^12^ Microvascular invasion was defined as second or above level of microvascular invasion and microscopic tumor thrombus. On the assumption that intrahepatic metastases were mainly a product of vascular invasion, patients with intrahepatic metastases were combined with patient group with microvascular invasion. Tumor stage was determined according to the tumor node metastasis (TNM) staging system, proposed by the American Joint Committee on Cancer (AJCC).

### Follow-Up and Survival Information

All patients were followed up regularly after discharge. Follow-up was scheduled monthly for the first 6 months, then trimonthly for the next 18 months and every half a year for the rest time until death or to October 30, 2014. Examinations consisted of ultrasonography, liver function, and AFP tests were performed at each time. Additional contrast-enhanced computed tomography (CT), magnetic resonance imaging (MRI), or hepatic angiography would be ordered if a recurrence or progression of the disease was suspected. Further therapeutic regimes such as local ablation, a second surgery, TACE, or molecular targeted therapy would be undertaken if disease relapse or progression was confirmed.

### Preoperative Imaging Classification

Each patient underwent plain and contrast-enhanced CT scans before surgery on a multidetector CT scanner (LightSpeed VCT, GE Healthcare, Milwaukee, WI, USA) with a slice thickness of 5.0 mm. Delay time was set as 30, 60, and 180 seconds for hepatic arterial, portal venous, and equilibrium phases, respectively, after injection of 80 mL Omnipaque (350 mg I/mL, GE Healthcare) at a rate of 3 mL/second.

CT images were retrospectively reviewed independently by 2 blinded readers who were unaware of the patients’ pathological and clinical information apart from the diagnosis of HCC. They discussed to reach a consensus if there was disagreement. We defined the imaging diagnostic criteria for contrast-enhanced CT as follows: type 1 (SN type) was round or oval with clear boundaries in the arterial and portal phases, some cases showed a pseudocapsule sign; type 2 (SNEG type) was round or oval with a clear edge, combined with focal protrusions; type 3 (CMN type) was lobulated with multinodular enhancement in the arterial phase, some lesions showed separations in the portal and equilibrium phases; type 4 (infiltrative type) was extremely irregular with clear margin, or blurred with fuzzy edge.

### Statistical Analysis

Percentage of each type was calculated. Different demographic characteristics and clinicopathologic variates among the 4 types were compared. One-way analysis of variance was performed in continuous data presented as mean ± standard deviation. Chi-square test was used for categorical variables. Disease-free and overall survival curves were generated with Kaplan–Meier method. Log-rank test was performed to compare survival of each type and for univariate analysis of prognostic factors. Survival related factors were determined by using Cox proportional hazards model. Typing accuracy of CT was calculated according to pathologic classification. SPSS 17.0 software (SPSS, Inc., Chicago, IL) was used for statistical analysis and α level was set at 0.05.

## RESULTS

### General Information

A total of 144 patients with solitary HCC were enrolled. There were 120 men and 24 women, with a mean age of 53 ± 12 years (range: 22 ∼ 87). The 144 HCCs were composed of SN (25/144, 17.4%), SNEG (34/144, 23.6%), CMN (33/144, 22.9%), and infiltrative types (52/144, 36.1%). Patients with infiltrative type (50.1 ± 10.6 years) were significantly younger than those with other 3 types (*P* < 0.05) (Table 1).

**TABLE 1:**
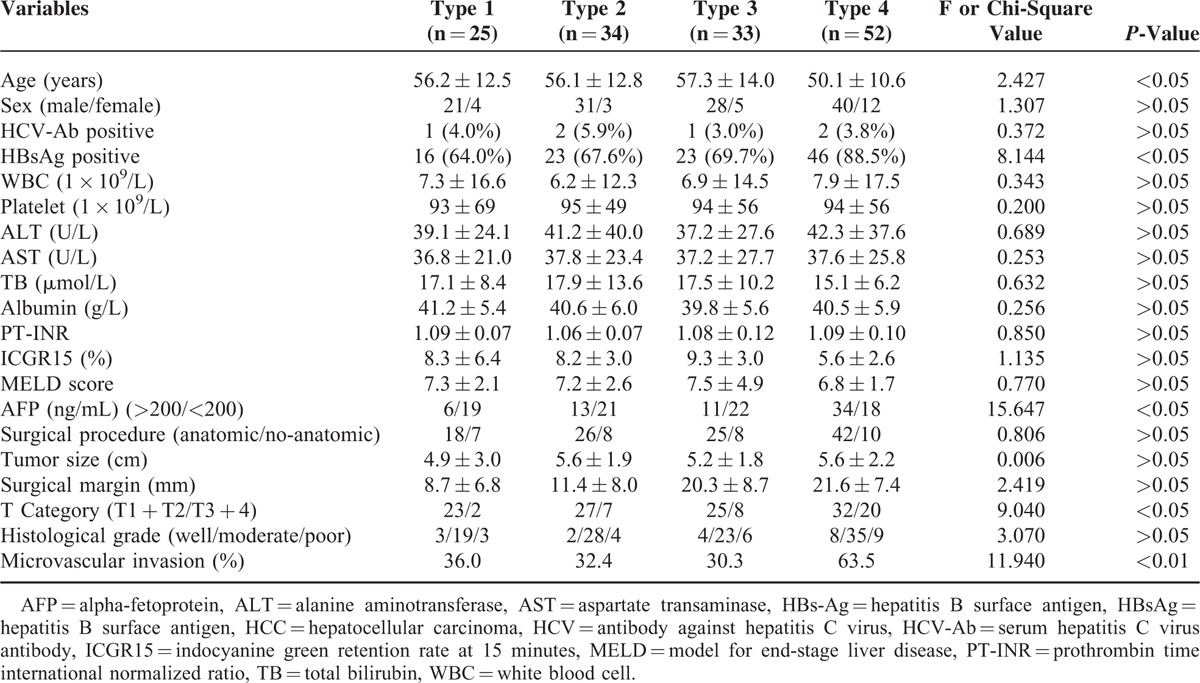
Comparison of Clinicopathological Variables of Patients With 4 Types of HCCs

### Clinicopathologic Results

The positive rate of serum HBsAg and preoperative serum AFP level in patients with infiltrative type were significantly higher than those with other 3 types (all *P* < 0.05). A total of 111 and 33 patients underwent anatomic and nonanatomic resections, respectively. Neither perioperative mortality nor serious complications were recorded in the patients. The microvascular invasion rate of infiltrative type was significantly higher than those of other 3 types (*P* < 0.05). There were 107 patients at stage T1 and T2, 37 at T3, and T4 according to AJCC. A higher percentage of T3 and T4 in patients with infiltrative type (20/52, 38.5%) were observed than other 3 types (*P* < 0.05) (Table 1).

### Follow-Up and Survival Information

**FIGURE 2:**
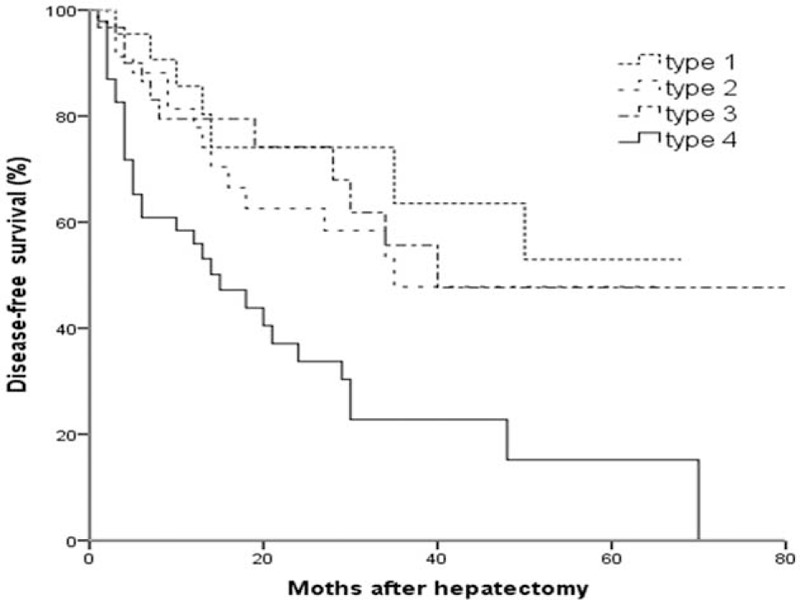
Kaplan–Meier curves for disease-free survival of patients with different gross types of solitary HCCs after hepatectomy.

The patients were followed up regularly with a median time of 26 months (range, 4 ∼ 74) and 3 patients were lost. Disease-free survival time of patients with infiltrative type (28.7 ± 5.4 months) was significantly shorter than other 3 types (47.6 ± 6.3, 45.6 ± 5.3, 42.3 ± 6.1, all *P* < 0.05) (Figure 2).

**FIGURE 3:**
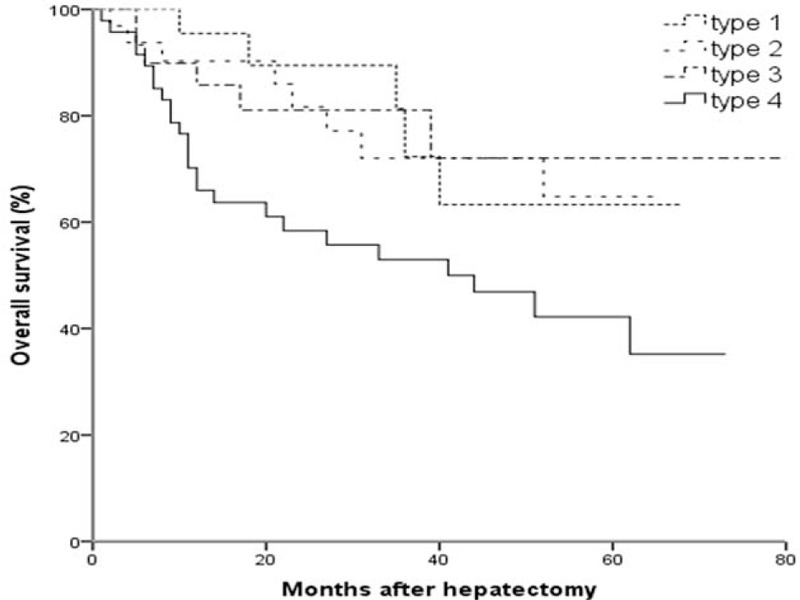
Kaplan–Meier curves for overall survival of patients with different gross types of solitary HCCs after hepatectomy.

**FIGURE 4:**
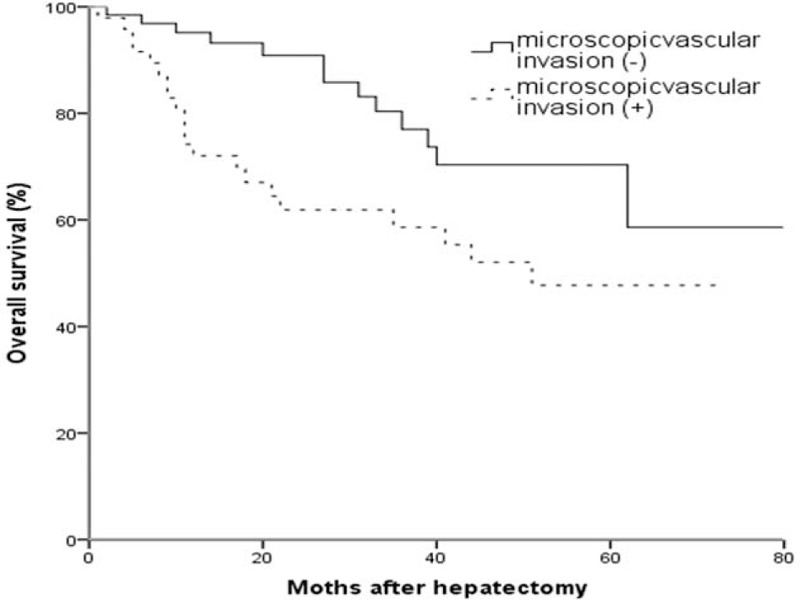
Kaplan–Meier curves for overall survival of patients with solitary HCCs with or without microvascular invasion.

Overall survival time of patients with infiltrative type (45.9 ± 5.1 months) was significantly shorter than other 3 types (56.3 ± 5.0, 56.4 ± 3.9, 53.3 ± 4.7, all *P* < 0.05) (Figure 3). Five-year survival rate was 55.6% (80/144) and 76.4% (110/144) in patients of HCC with and without microvascular invasion (*P* < 0.05) (Figure 4).

Univariate analysis showed that preoperative serum AFP, T stage of AJCC, microvascular invasion, and gross classification were risk factors of overall survival in patients with solitary HCCs. Multivariate analysis with Cox proportional hazards model showed that T stage of AJCC, microvascular invasion and gross classification were independent prognostic factors of patients with solitary HCCs (Table 2).

**TABLE 2:**
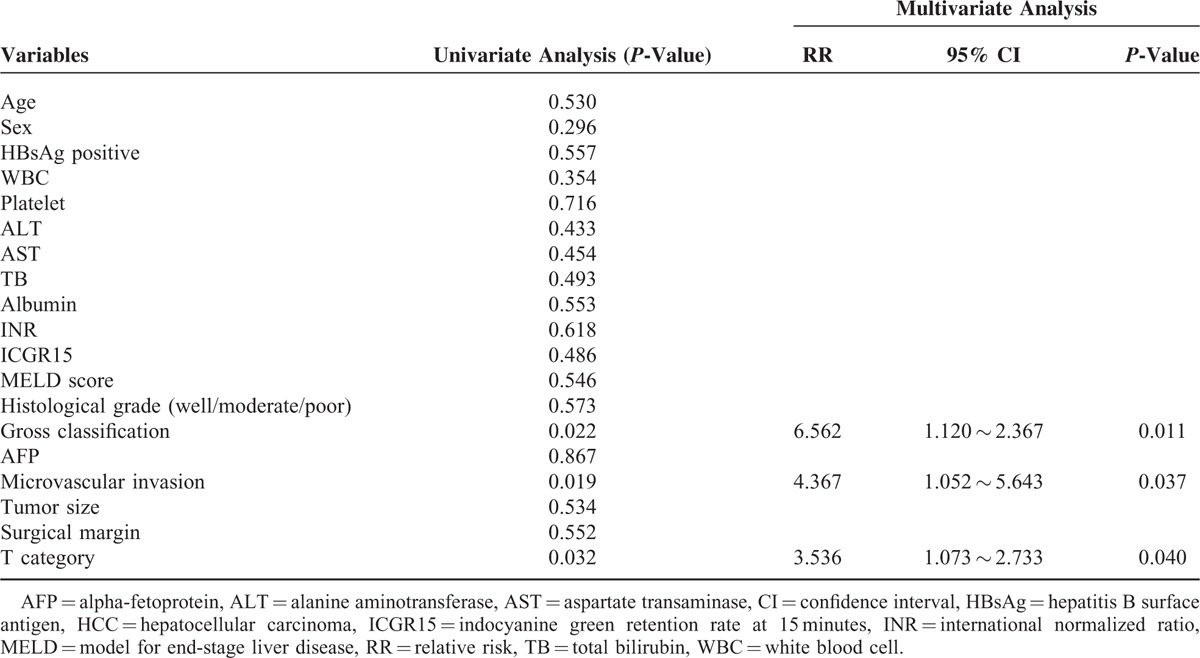
Univariate and Multivariate Analysis of Prognostic Factors of Patients With HCCs After Hepatectomy

### CT Classification Accuracy

Preoperative CT classification was coincident in 65.3% (94/144) of the patients. Identity reached 72.0% (18/25) in SN type, 35.3% (12/34) in SNEG type, 45.5% (15/33) in CMN type, and 59.6% (31/52) in infiltrative type, respectively.

## DISCUSSION

Eggel gross classification of HCC (nodular, massive, and diffuse) was introduced in 1901 based on autopsy reports.^1^ Kanai et al proposed a new gross classification in which Eggel nodular type was furtherly subclassified into type 1 (SN type), type 2 (SNEG type), type 3 (CNM type), and type 4 (poorly demarcated nodular type) based on surgically resected small HCCs (≤3 cm) data.^2^ Clinicopathologic features and prognostic significance of gross classification of HCC were generally investigated mainly on type 1 ∼ 3 but seldom on type 4 due to its low incidence and resection rate.^6–8,13^ Type 4 was also named invasive or infiltrative type. To our knowledge, this is the first study on clinicopathologic features and prognostic significance of gross classification in solitary HCCs including type 1 ∼ 4.

Based on the majority of previous reports, type 1 was most common (44 ∼ 80.4%), followed by type 2 (8.4 ∼ 31%), and 3 (9.9 ∼ 29.9%).^6,7,13,14^ However, Kanai et al^2^ and Yuki et al^15^ reported that type 2 was most common (34.4% and 78.2%) followed by type 3 (31.1% and 12.8%) and 1 (21.3% and 9.0%). Nevertheless, type 4 always occupied the lowest proportion (1.6 ∼ 18.0%). Our study found that type 4 ranked first (36.1%) followed by other 3 types (17.4 ∼ 23.6%), which proved our hypothesis.

In our cohort, the age of patients with type 4 (50.1 ± 10.6) was significantly younger than other 3 types (*P* < 0.05). Hui et al^6^ and Inayoshi et al^16^ reported that the age of patients with type 3 (51 ± 18, 62.2 ± 2.7 years) was significantly younger than those of type 1 and 2, while other authors reported no significant difference in age.^10,13^ Of note, we found that the ages of patients with type 1 and 2 in our study were younger than previous reports.^6,7,10,13,16^

Our cohort showed nearly the highest HBV (75.0%) and lowest HCV infection rate (4.2%) among previous reports.^2,6–8^ And the HBV infection rate in type 4 (88.5%) was significantly higher than other 3 types in our study. In previous studies, the HBV infection rate in type 3 was relatively higher (30 ∼ 41%) than type 1 and 2, and the HCV infection rate in type 2 (43 ∼ 100%) and 1 (62 ∼ 73%) was relatively higher than type 3 (40 ∼ 62.2%).^6–8^ Based on previous reports and our own data, we may deduce that type 4 and 3 are more related to HBV infection, while type 2 and 1 are associated with HCV infection. The serum AFP level in type 4 was significantly higher than other 3 types in our study. The highest serum AFP level was also reported in type 2 and 3 among type 1 ∼ 3.^7,8,13^ Anyway, the serum AFP level was usually the lowest in type 1 among all the type,^8,13^ which suggests that the serum AFP level might be helpful to differentiate boundary (type 1) and nonboundary types (type 2 ∼ 4).

The microvascular invasion rate of type 2 (25 ∼ 93.4%) and 3 HCC (12.5 ∼ 100%) were reported significantly higher than that of type 1 (7.7 ∼ 45.8%).^2,6–8,13–15^ In our study, the microvascular invasion rate of type 4 (63.5%) was significantly higher than other 3 types (30.3 ∼ 36%).

Due to a relatively larger tumor diameter and higher anatomic resection rate (92.3%) in patients with type 4 HCC, larger extent and longer margin of resection, more intraoperative blood loss and perioperative transfusion, longer operation, and hospitalization time were recorded in patients with type 4 HCC compared with other 3 types (all *P* > 0.05 except blood loss). Blood loss of our patients, which was comparable with Tsujita report,^7^ was much less than Ueno report.^14^ However, the mean resection margin (10.7 ± 11.6 mm) of patients with type 4 HCC in our study was shorter than Ueno report (16.8 ± 16.0 mm).^14^ And Ueno et al found that micrometastases in the nonboundary type (type 2 ∼ 4) were further from the main tumor (9.5 ± 6.2 mm) than those in the boundary type (type 1) (within 3.1 ± 1.4 mm).^14^ In patients with nonboundary type HCC, anatomic resection was recommended to the extent that liver function allows in order eradicating micrometastases that have extended away from the tumor's margin.^14^

Five-year survival rate of patients with type 4 (51.1%) after resection was first reported in our study, which was significantly lower than those of type 1 ∼ 3 (70 ∼ 80%). It was reported that 5-year survival rate of patients with type 1 after resection (50 ∼ 100%) was significantly better than those of type 2 (35 ∼ 71.6%) and 3 (35 ∼ 75%). A rough survival of our cohort (type 1 ∼ 3) was similar with that reported by Tsujita et al, ranging from 70 ∼ 80%.^7^ Tsujita and ours were much better than Shimada report^13^ in 2001, maybe owe to therapeutic advances during the last decade. However, survival of patients with type 1 and 2 after LDLT was as high as 90%, while that of type 3 was only 40%.^8^ To sum up, the most favorable treatment of patients with type 1 might be LDLT to erase underlying liver diseases completely, and then nonanatomic resection to preserve liver function as much as possible. For type 2, LDLT and anatomic resection could be chosen. The most effective therapy for patients with type 3 was anatomic resection while living donor-related liver transplantation was nearly a contradiction. Nevertheless, the most effective treatment for type 4 remains unknown.

Multivariate analysis in our study showed that gross classification, microvascular invasion and T stage were independent prognostic factors for overall survival after resection. Shimada et al^13^ reported that the presence of intrahepatic metastasis, the macroscopic classification of SNEG type, and absolute noncurative operation were independent poor indicators of prognosis by multivariate analysis. Hui et al^6^ reported that T category and gross classification were independent predictive variables of disease-specific survival. Choi et al^17^ reported that the gross classification of tumor type (single nodular vs. nonsingle nodular) was the only independent prognostic factor for both disease-free and overall survival after resection. Although there might be some relationship between gross classification and microvascular invasion, they are both independent prognostic factors. Poor prognosis of patients with type 4 HCC not only depended on its higher microvascular invasion rate, but also on its high potential of extra-hepatic invasion and unique molecular mechanisms.^8,16^

Hui et al^6^ reported a diagnostic accuracy of 46% for CT (slice thickness 10 mm) classification in HCC. By using thin-sliced CT (5 mm), dynamic CT with a large volume of contrast medium and establishing a reliable imaging criterion, the rate of correct diagnosis has been improved to 65% in our study. Hatanaka et al^18^ reported a similar result of 65.6% (40/61) for contrast-enhanced CT and a significant higher diagnostic accuracy of 86.9% (53/61) for contrast-enhanced ultrasonography. Tada et al^19^ reported that using Gd-EOB-DTPA-enhanced MRI to assess the macroscopic findings in nodular HCC was equal or superior to using angiography-assisted CT. The development of an objective morphological classification system using preoperative multiphase CT to preoperatively evaluate HCCs would cover the shortage of subjective classification.^20^ By combining imaging modalities such as preoperative contrast-enhanced ultrasonography, CT, MR, or even intraoperative ultrasonography,^18^ the gross classification of HCC may be mostly predicted. Our study showed that the preoperative gross classification based on contrast-enhanced CT had great prognostic significance in solitary small HCC treated with radiofrequency ablation.^21^ Gross classification before and during surgery is probably helpful in selecting the surgical procedure. Therefore, a highly malignant subclass of type 3 or 4 HCC, may be given a new treatment strategy such as anatomic resection to improve the outcome.

There were some limitations in our study. Firstly, the sample size was relatively small due to a single-institution study and the follow-up time was relatively short. Secondly, it remains unknown which is most likely to remove microvascular invasion: a wide surgical margin or anatomic resection? Thirdly, the classification accuracy of preoperative imaging was still low even with some improvement. All those issues require further studies.

In conclusion, we first reported that the incidence of type 4 HCC was significantly higher in Chinese patients compared with western and Japanese patients. And this type HCC was distinctively associated with higher HBV infection rate, lower presence of background liver disease, younger age, higher serum AFP level, poorer differentiated histology, higher microvascular invasion rate, and poorer survival rate. Anatomic resection was recommended in this type to eradicate microvascular invasion as much as possible.
